# Extracellular Neuroglobin as a Stress-Induced Factor Activating Pre-Adaptation Mechanisms against Oxidative Stress and Chemotherapy-Induced Cell Death in Breast Cancer

**DOI:** 10.3390/cancers12092451

**Published:** 2020-08-29

**Authors:** Marco Fiocchetti, Virginia Solar Fernandez, Marco Segatto, Stefano Leone, Paolo Cercola, Annalisa Massari, Francesco Cavaliere, Maria Marino

**Affiliations:** 1 Department of Science, University Roma Tre, Viale Guglielmo Marconi 446, I-00146 Roma, Italy; virginia.solarfernandez@uniroma3.it (V.S.F.); stefano.leone@uniroma3.it (S.L.); 2 Department of Biosciences and Territory, University of Molise, Contrada Fonte Lappone, 86090 Pesche (IS), Italy; marco.segatto@unimol.it; 3 Division of Senology, Belcolle Hospital, Str. Sammartinese, 01100 Viterbo, Italy; paolo.cercola@asl.vt.it (P.C.); annalisa.massari@asl.vt.it (A.M.); francesco.cavaliere@asl.vt.it (F.C.)

**Keywords:** 17β-Estradiol, apoptosis, breast cancer, docetaxel, neuroglobin, oxidative stress, stress adaptation and resistance, tumor microenvironment

## Abstract

Components of tumor microenvironment, including tumor and/or stromal cells-derived factors, exert a critical role in breast cancer (BC) progression. Here we evaluated the possible role of neuroglobin (NGB), a monomeric globin that acts as a compensatory protein against oxidative and apoptotic processes, as part of BC microenvironment. The extracellular NGB levels were evaluated by immunofluorescence of BC tissue sections and by Western blot of the culture media of BC cell lines. Moreover, reactive oxygen species (ROS) generation, cell apoptosis, and cell migration were evaluated in different BC cells and non-tumorigenic epithelial mammary cells treated with BC cells (i.e., Michigan Cancer Foundation-7, MCF-7) conditioned culture media and extracellular NGB. Results demonstrate that NGB is a component of BC microenvironment. NGB is released in tumor microenvironment by BC cells only under oxidative stress conditions where it can act as autocrine/paracrine factor able to communicate cell resilience against oxidative stress and chemotherapeutic treatment.

## 1. Introduction

Tumor microenvironment encompasses a complex mixture of cancer and non-cancerous cells (i.e., immune and endothelial cells, fibroblasts, adipocytes) and the extracellular matrix (ECM) [1,2,3]. A binary intertwined connection exists between all the components of tumor microenvironment as demonstrated in breast cancer (BC) [2,3]. On one side, tumor cells directly interact each other and sculpt the ECM by releasing a variety of proteins that act as autocrine or paracrine factors influencing cancer and non-cancer cell proliferation, differentiation, and survival [1]. On the other hand, released factors from cancer-associated fibroblasts (CAF) and adipocytes (CAA) contribute to the tumorigenic phenotype of pre-malignant and malignant epithelial cells [4] promoting tumor cell proliferation, invasiveness, and growth. In addition, CAF-released factors influence the development of therapy resistance by inducing anti-apoptotic mechanisms in both estrogen receptor α positive (ERα+) and negative (ERα−) breast cancer cells exposed to anti-cancer drugs [5,6]. Moreover, the direct role of BC-associated fibroblasts in reducing hormone sensitivity and increasing resistance to endocrine therapy (i.e., Tamoxifen) has been demonstrated in ERα+ breast cancer cells [2,6]. The effect of heterotypic interaction between epithelial breast cancer cells and non-cancerous stromal cells have attracted major research interests focused in the discovery of new druggable pathways involved in cancer cell adaptation/resistance to stressful conditions and therapeutic strategies [1,2,4]. However, the identification of molecular factors and mechanisms by which cancer cells may affect the extracellular milieu and, in turn, neighbor normal and/or cancer epithelial cells in homotypic way is still in its infancy. Among different factors, including growth factors, cytokines, chemokines, and microRNAs, released by breast cancer cells to cope with stressful conditions altering the tumor microenvironment [1], the 17β-Estradiol (E2)-inducible compensatory protein named neuroglobin (NGB) [7] deserves particular consideration for its pro-survival and anti-apoptotic role in BC [8]. Indeed, in ERα+ breast cancer cells, high level of E2-induced NGB takes part in the ERα-dependent pathways committed to the anti-apoptotic response against oxidative stress [7] and paclitaxel, a chemotherapeutic agent [9]; moreover, NGB participates in the E2-induced anti-oxidant effects through the NRF-2 pathway [10]. Recently, NGB was found in astrocyte-derived exosomes [11], and treatment with sub-nanomolar concentration of extracellular NGB induces a protective effect against oxidative stress and apoptotic cell death in astroglial cells [12]. These data sustain the idea that NGB could be released in the extracellular milieu where the globin could elicit its pro-survival functions outside the cells. Whether NGB may play a similar extracellular role in BC is completely unknown. The present study is aimed at evaluating the possibility that E2 and/or hydrogen peroxide (H_2_O_2_) could spread NGB outside the cells acting as inter-cellular factor involved in the breast cancer cell adaptation and survival to stress. Human tissues, ERα+/− breast cancer cells, and non-tumorigenic epithelial mammary cells were used as experimental models.

## 2. Results

### 2.1. Analysis of NGB Extracellular Release by Bio-Informatic Approaches and in BC Tissue

**Figure 1:**
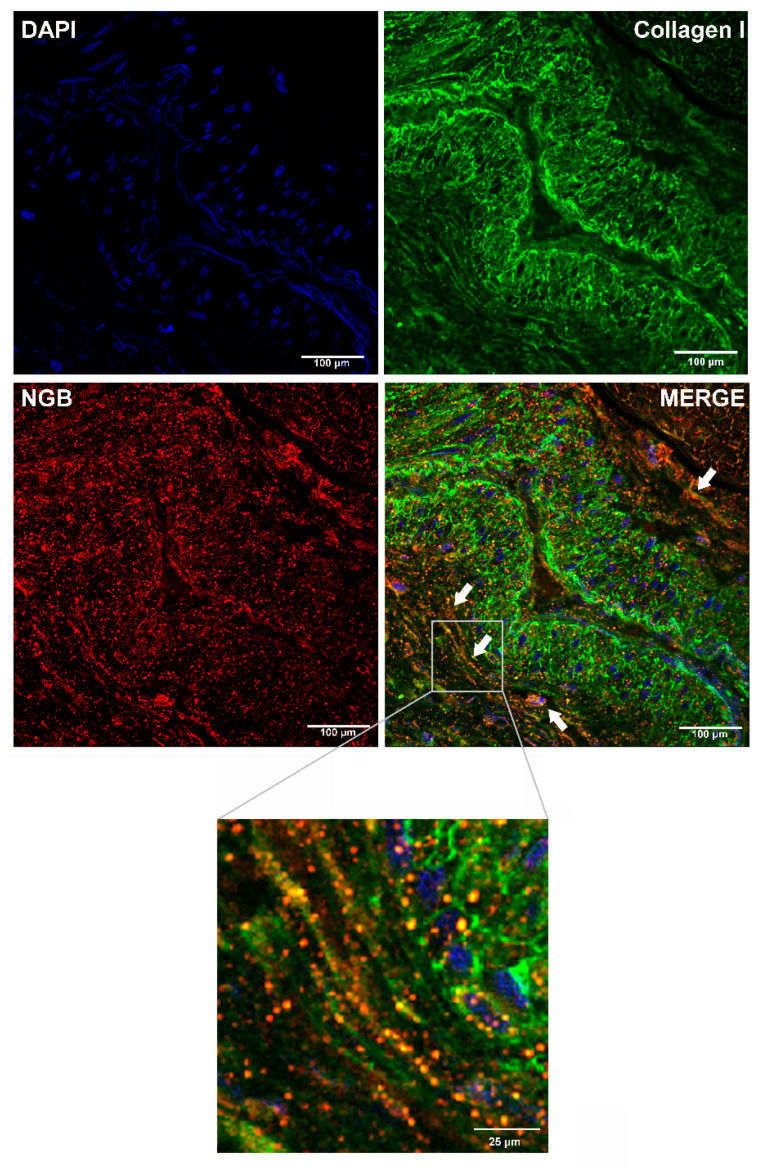
In vivo neuroglobin (NGB) extracellular localization. Confocal microscopy analysis of NGB and collagen I co-immuno-localization in human breast cancer tissue (ERα+; grade G2) sections. The sections were de-paraffinized, boiled in a microwave for antigen retrieval, blocked with 3% Bovine Serum Albumine (BSA) in Phosphate Buffered Saline (PBS) + Triton-X 100 0.5%, stained with 4’,6-Diamidino-2-Phenylindole (DAPI) for nuclei (blue), the anti-NGB (red) and anti-Collagen I antibodies (green) (original magnification 40×). Co-immunolocalization between NGB and collagen I fibers (yellow signals) is indicated by white arrows in merged image. The gray square refers to merged image detail reported on the bottom as a digital magnification. The scale bars are 100 µm/cm in the first 4 panels and 25 µm/cm in the enlarged panel at the bottom. All images are single planes and are representative of 10 independent experiments.

The possibility that NGB could be released in the extracellular milieu was firstly evaluated by bioinformatic approaches analyzing the NGB primary sequence for prediction of extracellular secretion via the conventional (SignalP 5.0; [13]) or unconventional (SecretomeP 1.0; [14]) pathways. Obtained data from SignalP 5.0 indicate a very low probability (likelihood = 0.000944) that NGB sequence contains any “standard” secretory signal peptides, ruling out the possibilities that NGB can be properly secreted through the conventional secretory pathway. Differently, the high NN (Neuronal Network) score in SecretomeP 1.0 for NGB (0.913; NN limit threshold = 0.6) indicates that the protein could be potentially secreted via an unconventional secretory pathway, sustaining the hypothesis that NGB could be released outside cells. The bioinformatic analyses prompted us to confirm the rising hypothesis in human specimens by using paraffin-embedded sections of ERα+ invasive ductal carcinoma grade G2 from at least 10 breast cancer patients. The confocal microscopy analysis reveals the co-immunolocalization of NGB with collagen I, the most abundant collagen in ECM of tumors including BC [15,16], principally at the level of the extracellular matrix as indicated by the absence of any nuclear DAPI signals (Figure 1; merged image; white arrows and square of merge image detail), strengthening the idea of an in vivo extracellular NGB localization.

### 2.2. Effect of E2 and H_2_O_2_ on NGB Release from MCF-7 Cells

**Figure 2:**
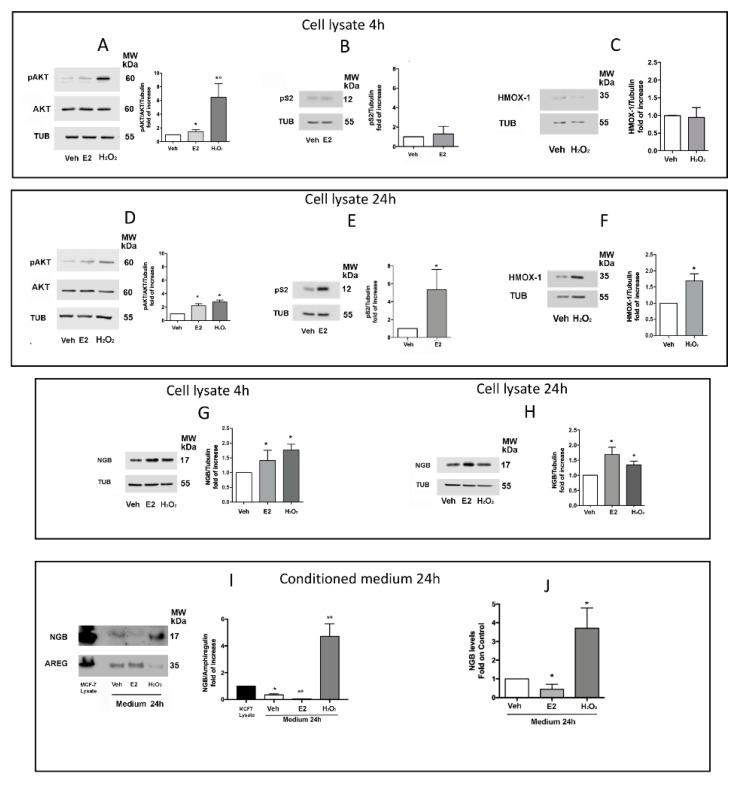
Effect of E2 and H_2_O_2_ stimulation on intracellular and extracellular NGB levels. Western blot images (left) and densitometric analysis (right) of intracellular (cell lysate) levels of pAKT(S473) (**A**,**D**), pS2 (**B**,**E**), HMOX-1 (**C**,**F**), and NGB (**G**,**H**) in MCF-7 treated with vehicle (EtOH/PBS 1/10 *v/v*), E2 (10 nM, 30 min), or H_2_O_2_ (200 μM; 30 min) as reported in Figure S1 and harvested 4 or 24 h after cell washing. The amount of protein was normalized by comparison with tubulin levels or with total AKT and tubulin levels (**A**,**D**). Western Blot representative images of NGB protein levels in conditioned media generated by E2 or H_2_O_2_ treated cells after 24 h of cell culturing (**I**). The level of NGB in MCF-7 cell lysates was used as positive control of NGB expression. The amount of protein was normalized by comparison with amphiregulin (AREG) protein levels. Evaluation of extracellular NGB levels through ELISA sandwich analysis in 24 h conditioned media (**J**). Data are means ± SD of at least three different experiments. *p* < 0.01 was determined with Student’s *t*-test vs. Veh condition (*) and vs. E2 treatment (°) (**A**). The whole blot images can be found in Figure S2.

Previous results indicated that NGB intracellular levels and localization are positively modulated in a panel of ERα+ breast cancer cells (MCF-7, ZR-751, T47D) either by E2, oxidative stress (H_2_O_2_), and reactive oxygen species (ROS)-inducing compounds [7,8,17]. Based on the idea that any stimulus able to upregulate NGB changing its localization at the intracellular levels could also modify NGB levels outside cells, the effect of E2 (10 nM) or H_2_O_2_ (200 μM) on NGB release from MCF-7 cells was evaluated. In particular, applying the experimental protocol reported in Section 4 (Figure S1), MCF-7 cells were treated with Vehicle (EtOH/PBS, 1/10, v/v) or E2 (10 nM) or H_2_O_2_ (200 μM) for 30 min and, after washing and medium changing, cells were kept in culture for further 4 or 24 h to obtain the corresponding cell lysates (Veh; E2; H_2_O_2_) and conditioned media (CM-Veh; CM-E2; CM-H_2_O_2_). In order to validate the experimental protocol (Figure S1), the rapid activation of AKT (phosphorylation at S473) as well as the levels of pS2 and HMOX-1 (heme oxygenase-decycling-1) proteins, which respectively represent well known rapid (4 h) and long term (24 h) function regulated by E2 or H_2_O_2_ [17,18,19,20,21,22], were analyzed in cell lysates. As reported in Figure 2, both E2 and H_2_O_2_ induce AKT phosphorylation (Figure 2A,D). Remarkably, H_2_O_2_ stimulation rapidly (4 h) increases AKT phosphorylation (6.5 ± 1.6 fold on control) that decreases along time (24 h; 2.7 ± 0.2 fold on control), while E2 stimulation induces a less strong (1.5 ± 0.2 fold on control) (Figure 2A) but persistent activation of AKT (2.2 ± 0.2 fold on control) (Figure 2D), suggesting that, in accordance with our previous studies [17], E2 and oxidative stress activate divergent AKT pathways with differences in timing and intensity. On the other side, pS2 and HMOX-1 protein levels are, respectively, enhanced by E2 and H_2_O_2_ only after 24 h (Figure 2E,F), confirming that both E2- and H_2_O_2_-induced transcriptional mechanisms require more than 4 h to induce protein accumulation. Successively, the impact of E2 and H_2_O_2_ treatment on NGB levels in cell lysate and CM was evaluated. In accordance to previous studies [7,8,17], NGB intracellular levels significantly increase 4 h after E2 and H_2_O_2_ treatment (Figure 2G). Although both stimuli maintain high protein levels at 24 h of cell culturing (Figure 2H), the Western blot analysis of NGB in CM with respect to amphiregulin (AREG), a commonly released factor by ERα+ breast cancer cells [23], and the ELISA analysis of CM indicate that H_2_O_2_ greatly accumulates NGB in the extracellular milieu, while E2 triggers a significant reduction to the control (Figure 2I,J), indicating that the two NGB inducers exert opposite effects on NGB extracellular release.

### 2.3. Effects of Extracellular NGB on Breast Cancer and Non-Tumorigenic Mammary Epithelial Cells

**Figure 3:**
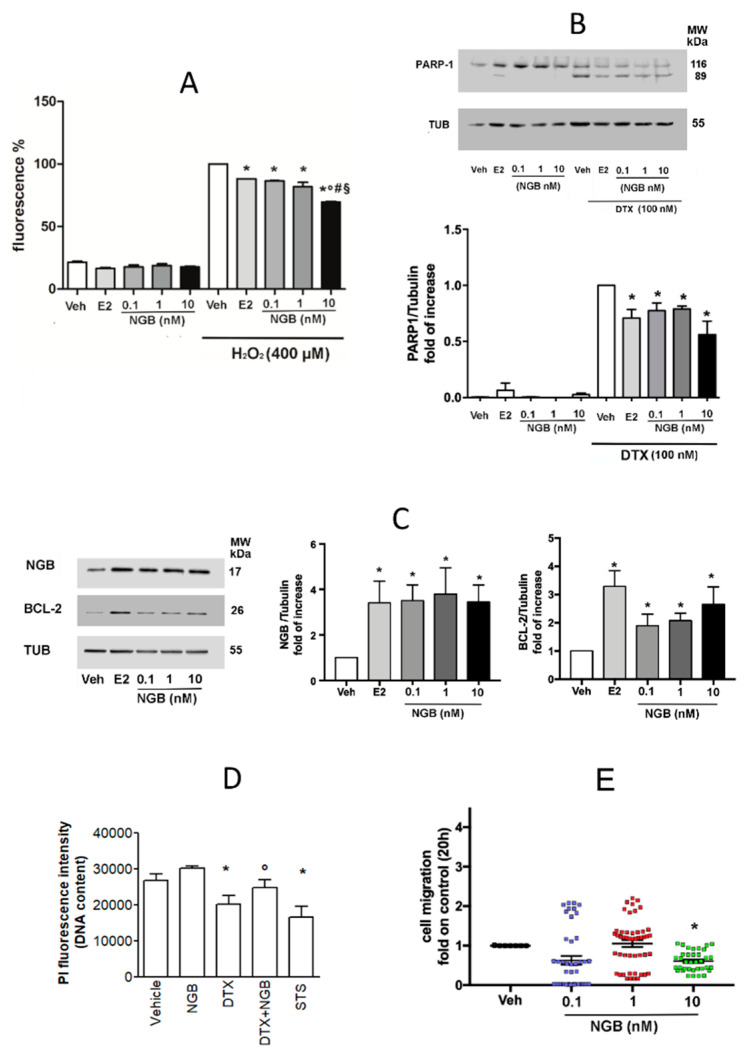
Effects of exogenous NGB on MCF-7 cell phenotype. (**A**) Reactive oxygen species (ROS) generation in MCF-7 cells pretreated with vehicle (EtOH/PBS 1/10 *v/v*), E2 (10 nM), or exogenous NGB (0.1, 1, and 10 nM) for 4 h and then exposed to H_2_O_2_ (400 μM; 30 min). Data are shown as percentage with respect to H_2_O_2_ treatment alone (100%). (**B**) Western blot (upper panel) and densitometric analyses (bottom panel) of PARP-1 cleavage in MCF-7 stimulated with vehicle (EtOH/PBS 1/10 *v/v*), E2 (10 nM) or exogenous NGB (0.1, 1, and 10 nM) (4 h) in presence or absence of following apoptotic stimulation with docetaxel (DTX; 100 nM; 48 h). (**C**) Representative Western blot (left) and densitometric analyses (right) of intracellular NGB and Bcl-2 protein levels in MCF-7 cells treated with vehicle (EtOH/PBS 1/10 *v/v*), E2 (10 nM) or exogenous NGB (0.1, 1, and 10 nM) for 48 h. The amount of protein was normalized by comparison with tubulin levels. (**D**) Analyses of MCF-7 cell DNA content obtained from propidium iodine assay (PI). Cells were stimulated with vehicle (EtOH/PBS 1/10 *v/v*) and exogenous NGB (10 nM) (4 h) in presence or absence of following apoptotic stimulation with docetaxel (DTX; 100 nM; 48 h). Data are means ± SD of at least three different experiments. *p* < 0.01 was determined with ANOVA followed by Tukey-Kramer post-test vs. Vehicle (*) or docetaxel (°) treatment. (**E**) Cell migration analysis of MCF-7 treated with vehicle (EtOH/PBS 1/10 *v/v*) or NGB (0.1, 1, and 10 nM) for 20 h. Data are means ± SD of at least three different experiments. *p* < 0.01 was determined with ANOVA followed by Tukey–Kramer post-test vs. Veh-H_2_O_2_ condition (*), vs. E2-H_2_O_2_ condition (°), vs. 0.1 nM NGB-H_2_O_2_ condition (#), vs. 1 nM NGB-H_2_O_2_ condition (§) (**A**) or Student’s *t*-test vs. Veh-DTX condition (*) (**B**) or vs. Veh treatment (*) (**C**,**E**). The whole blot images can be found in Figure S3.

The obtained data prompted us to evaluate the possible effects of extracellular NGB on breast cancer cells, mainly focusing on cell adaptation to oxidative stress, survival, and migration. MCF-7 cells were pretreated with nanomolar and sub-nanomolar NGB protein concentration in a dose-response curve (0.1, 1, and 10 nM; 4 h) and exposed to high dose of H_2_O_2_ (400 µM; 30 min). E2 pretreatment (10 nM; 4 h) was used as positive control. NGB stimulation significantly reduces the amount of intracellular ROS levels after cell exposure to H_2_O_2_, without affecting the basal level of ROS in absence of H_2_O_2_ stimulation (Figure 3A). The key role played by intracellular NGB in enhancing the resistance of breast cancer cells to taxane chemotherapeutic drugs (i.e., Paclitaxel [9]) prompted us to test the efficacy of NGB extracellular stimulation in inducing pro-survival effects in MCF-7 cells treated with docetaxel (DTX), a cytotoxic taxane commonly preferred in clinical practice [24]. Figure 3B shows that all NGB concentrations tested significantly reduce the levels of cleaved PARP-1 (89 KDa band), a common marker of apoptosis, induced by DTX (100 nM; 48 h) treatment. In addition, NGB stimulation significantly upregulates the protein levels of the anti-apoptotic proteins Bcl-2 [25,26] and of NGB itself [7,17], similarly to the positive control E2 (Figure 3C). This result has been further confirmed by the propidium iodine (PI) assay, which allows determining cell vitality. As expected, DTX treatment (100 nM) reduces the MCF-7 cell number; notably, NGB pretreatment abolished the DTX effect maintaining the cell number close to the control (Figure 3D). Then, MCF-7 cells were treated with different concentrations of NGB and cell migration was assessed. As reported in Figure 3E neither 0.1 nor 1 nM of NGB concentrations affect cell motility, whereas high NGB concentration (10 nM) significantly slows down MCF-7 migration, supporting the notion that extracellular NGB could direct cancer cell response mainly toward a proper adaptation to stress conditions.

**Figure 4:**
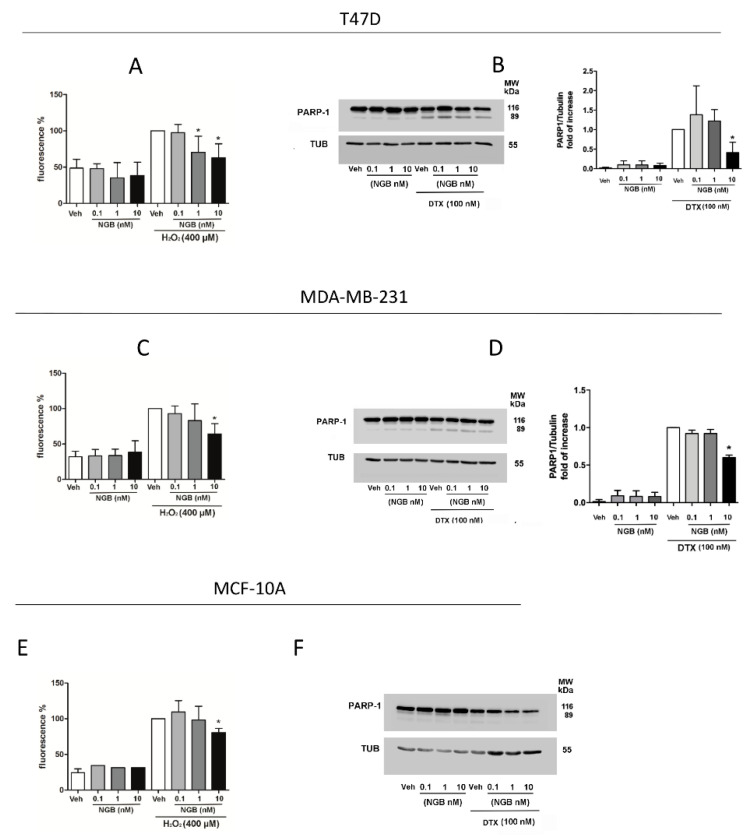
Effects of exogenous NGB on ERα +/− breast cancer and non-tumorigenic epithelial mammary cells. 2′,7′-Dichlorofluorescin diacetate (DCFHA-DA) analysis of ROS production in ERα+ T47D (**A**) ERα-MDA-MB-231 (**C**) breast cancer cells and non-tumorigenic epithelial mammary cells MCF-10A (**E**) pretreated for 4 h with a dose-curve of exogenous NGB (0.1, 1, and 10 nM) and then exposed to H_2_O_2_ (400 μM; 30 min). Data are shown as percentage respect to H_2_O_2_ treatment alone (100%). Analysis of PARP-1 cleavage in T47D (**B**), MDA-MB-231 (**D**), and MCF-10A (**F**) treated with exogenous NGB (0.1, 1, and 10 nM; 4 h pretreatment) in presence or absence of docetaxel (DTX; 100 nM; 48 h). The amount of protein was normalized by comparison with tubulin levels. Representative Western blots (left **B**,**D**,**F**) and densitometric analysis (right **B**,**D**) are reported. Data are means ± SD of at least three different experiments. *p* < 0.01 was determined with ANOVA followed by Tukey-Kramer post-test vs. Veh-H_2_O_2_ treatment (*) (**A**,**C**,**E**) or Student’s *t*-test vs. Veh-DTX (**B**,**D**) condition (*). The whole blots images can be found in Figure S4.

The role played by extracellular NGB in enhancing MCF-7 cell antioxidant and pro-survival response has been verified in other ERα+ (T47D) and ERα- (MDA-MB-231) breast cancer cell lines as well as in non-tumorigenic epithelial mammary cells (MCF-10A). Remarkably, NGB treatment lowers oxidative stress upon H_2_O_2_ (400 µM; 30 min) stimulation, and PARP-1 cleavage under DTX treatment (100 nM; 48 h) in all breast cancer cell lines considered (Figure 4), although significant effects were observed at higher globin concentrations than in MCF-7 cell lines. In particular, 10 nM of NGB significantly diminishes the H_2_O_2_-induced oxidative stress in MDA-MB-231 (Figure 4C) and prevents the DTX-induced PARP1-cleavage in T47D and MDA-MB-231 cells (Figure 4B,D). Differently, the effect of NGB treatment on intracellular ROS production starts at 1 nM in T47D cells and is maintained at the high concentration of 10 nM (Figure 4A). Of note, high NGB extracellular concentration (10 nM) also reduces the H_2_O_2_-induced oxidative stress in MCF-10A cells (Figure 4E) which, however, show low susceptibility to DTX-induced apoptotic cell death (Figure 4F).

### 2.4. Effects of Homotypic MCF-7 Derived Conditioned Media on Breast Cancer Phenotype

**Figure 5:**
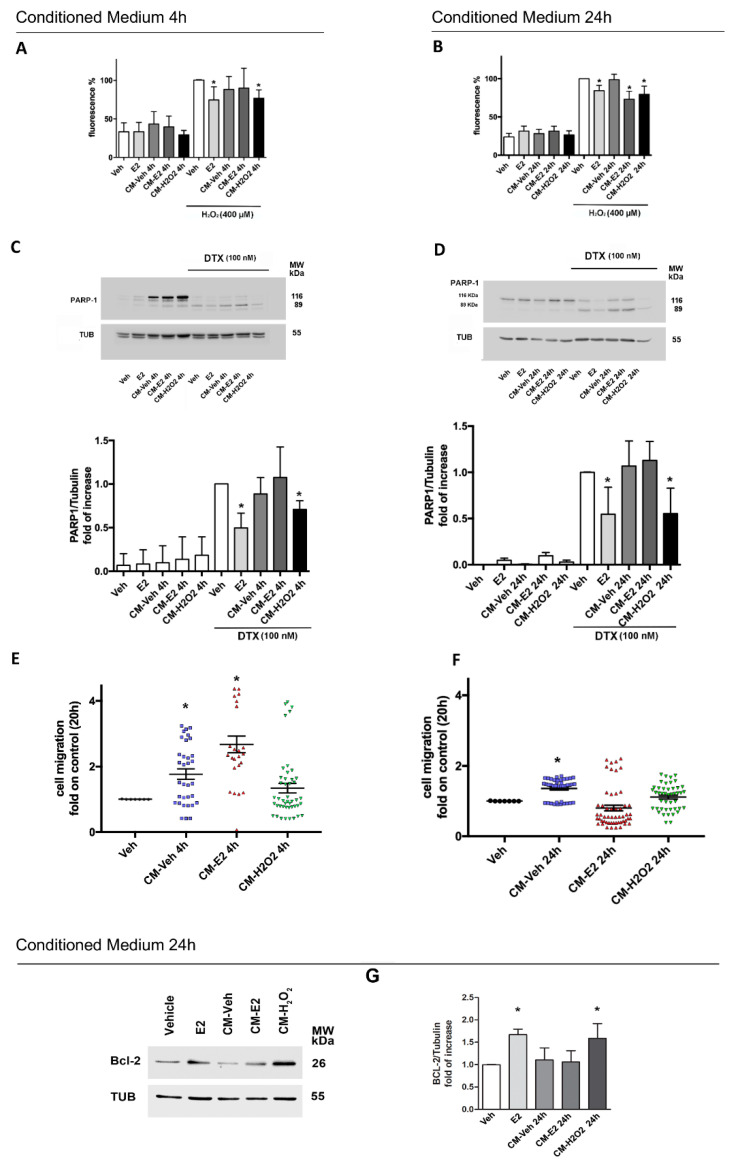
Effects of homotypic conditioned medium on MCF-7 cells phenotype. MCF-7 cells were treated in homotypic way with conditioned media obtained as reported in Figure S1 from MCF-7 cells stimulated with vehicle (EtOH/PBS 1/10 *v/v*), E2 (10 nM, 30 min), or H_2_O_2_ (200 μM; 30 min) and further cultured for 4 h (CM-Veh 4 h, CM-E2 4 h, CM-H_2_O_2_ 4 h; left side) or 24 h (CM-Veh 24 h, CM-E2 24 h, CM-H_2_O_2_ 24 h; right side). Effects of 4 h pretreatment conditioned media 4 h (CM 4 h) or 24 h (CM 24 h) on ROS generation in presence or absence of cells exposure to H_2_O_2_ (400 μM; 30 min) (**A**,**B**) and on PARP-1 cleavage in presence or absence of DTX stimulation (100 nM; 48 h) (**C**,**D**). E2 (10 nM; 4 h) pretreatment was used as positive control. In PARP-1 analysis, the amount of protein was normalized by comparison with tubulin levels. Upper panels are representative Western blots and bottom panels are corresponding densitometric analysis (**C**,**D**). Cell migration analysis of MCF-7 treated with conditioned media 4 h (**E**) or 24 h (**F**) for 20 h. Bcl-2 levels in MCF-7 treated with E2 (10 nM, 30 min), or conditioned media 24 h; left panel is the representative Western blot, right panel are the corresponding densitometric analysis (**G**). Data are means ± SD of at least three different experiments. *p* < 0.01 was determined with ANOVA followed by Tukey-Kramer post-test vs. Veh-H_2_O_2_ treated samples (*) (**A**,**B**) or Student’s *t*-test vs. Veh-DTX conditions (*) (**C**,**D**) or Veh treatment alone (*) (**E**,**F**,**G**). The whole blots images can be found in Figure S5.

The effects of MCF-7 derived conditioned media, obtained as reported in Figure S1, have been assessed on H_2_O_2_-induced ROS production, DTX-induced PARP-1 cleavage, and cell migration. As reported in Figure 5, MCF-7 cells treatment with homotypic CM generated from cells treated with H_2_O_2_ and kept in culture for 4 h (CM-H_2_O_2_ 4 h) or 24 h (CM-H_2_O_2_ 24 h) significantly reduce the H_2_O_2_-induced oxidative stress (Figure 5A,B) and decrease PARP-1 cleavage mediated by DTX stimulation (Figure 5C,D). Neither CM-Veh 4 h nor CM-Veh 24 h or CM-E2 4 h have any effect on ROS generation or apoptotic cell death induced by DTX (Figure 5A–D). However, the CM obtained from MCF-7 cells treated with E2 (10 nM; 30 min) and kept in culture for 24 h (CM-E2 24 h) lowers the oxidative stress upon high dose of H_2_O_2_ (Figure 5B), but it is ineffective in reducing DTX-induced PARP-1 cleavage (Figure 5D). On the other hand, CM-Veh at both 4 and 24 h, and CM-E2 4 h significantly promotes cell motility, whereas CM-E2 24 h and CM-H_2_O_2_ obtained at both 4 and 24 h do not affect migration (Figure 5E,F). These data suggest that both short-term and long-term response to H_2_O_2_ (CM-H_2_O_2_ 4 h; CM-H_2_O_2_ 24 h) as well as long-term effect of E2 (CM-E2 24 h) modify the composition of conditioned medium rendering it able to counteract pro-migratory factors released by cells under resting conditions (CM-Veh 4 h, CM-Veh 24 h). As the extracellular NGB increase the level of the antiapoptotic protein Bcl-2 (Figure 3C) and high NGB levels were found in CM-H_2_O_2_ 24 h, the possible effect of 24 h CMs on Bcl-2 levels was evaluated. Figure 5G shows that, apart the positive control (i.e., E2), just CM-H_2_O_2_ significantly induces Bcl-2 accumulation sustaining the effects of extracellular NGB treatment.

## 3. Discussion

NGB is a small intracellular monomeric globin, firstly discovered in neurons of central and peripheral nervous system [27]. Over the years, a key neuroprotective effect has been ascribed to the overexpressed NGB in neurons against several types of insults (i.e., hypoxia, oxidative stress, oxygen/glucose deprivation) [28,29,30,31,32,33,34,35]. Furthermore, recent results have clearly indicated that high level of intracellular NGB protein exerts a critical role in the E2, ERα-dependent, antioxidant, and pro-survival effects on breast cancer cells [7,10,17].

Remarkably, almost 20 years of scientific research have given a plethora of possible fascinating functions of NGB in both neurons and extra-nervous cancer cells that are defined not only by its whole cell concentration, but also by its intracellular compartmentalization [8,33,36]. Indeed, during the last years, the idea that NGB localization may provide some clues about its function and that stimuli able to change NGB level/localization might also affect such function, has earned growing interest [8,33,36,37,38]. Other independent studies further complicate this scenario, indicating that NGB may be released from astrocytes [11] and exogenous NGB can exert cytoprotective functions in astroglial cells [12] and retinal neurons [39].

In this context, here, we were aimed at investigating the possibility that NGB could be extracellularly released by breast cancer cells to exert putative exogenous functions on cancer cells phenotype. Data reported indicate the presence of NGB in the extracellular matrix of breast cancer sections derived from human patients with ERα+ Grade 2 ductal carcinoma. A great amount of globin co-localizes with collagen fibers, suggesting that NGB can be released by breast cancer cells in vivo. This result, in line with evidence demonstrating the presence of NGB in astrocytes derived exosomes [11], in serum of animal models after ischemic-reperfusion insults [40], and in plasma of human patients with traumatic brain injury [41], indicates that the extracellular NGB release is a common feature of this globin.

At cellular level, accordingly to our previous studies indicating the role of intracellular NGB as an oxidative stress sensor [8,17], H_2_O_2_ increases endogenous NGB levels. In parallel, we provided the first evidence that the same treatment with a pulse of H_2_O_2_ promotes NGB accumulation in the extracellular milieu 24 h after cell culturing. On the contrary, E2 stimulation, which increases intracellular NGB in a similar fashion to H_2_O_2_, significantly reduces NGB levels in the extracellular compartment with respect to the control. As previously reported, E2 increases NGB intracellular levels and promotes the globin shuttling and gathering into mitochondria, where NGB acts as a negative regulator of the intrinsic apoptotic pathway [7,17]. On the other side, both H_2_O_2_ and ROS inducing compounds (i.e., Lead Acetate) increase the intracellular NGB protein levels mainly at the cytosolic compartment [8,17]. When evaluated as a whole, these data strongly sustain the idea that diverse stimuli may differently modulate NGB intracellular/extracellular localization. In particular, the hormone-activated pathways preferentially re-allocate NGB at the intracellular compartments preventing the extracellular release of globin, whereas oxidative stress promotes NGB accumulation in cytosol that precedes the subsequent protein release out of the cells.

Soluble secreted proteins commonly contain *N*-terminal signal peptide or hydrophobic sequence that drive them to endoplasmic reticulum/Golgi network to be released extracellularly after fusion of Golgi derived vesicle with plasma membrane [42,43]. Beside this classical secretion pathway [44,45], several other cytoplasmic proteins follow alternative mechanisms of secretion collectively defined as non-classical or unconventional secretory pathways (UPS) [42,44,46] commonly activated under stressed conditions [46,47]. Here, we reported that the probability (SignalP 5.0 [13]) of NGB sequence to contain any *N*-terminal signal that could direct it toward the classical secretory pathway is very low. In parallel, we applied another sequence-based method (Secretome P) which evaluates several protein features that are commonly shared between the well-known non-classical secretory proteins (i.e., FGF-1, FGF-2, IL-1B) [14] to predict protein secretion through the UPS. The high neural network score of NGB indicates a positive prediction for the globin to be secreted through a non-classical mechanism. At least four types of non-classical protein secretion have been distinguished and they comprise the direct translocation across the membrane (Type I and Type II), the organelle-based translocation (autophagosome, endosome, and exosomes, Type III) and the ER-based translocation with Golgi bypass (Type IV) [42,43,46]. Although NGB may follow one or more of the different mechanisms of non-classical protein secretion, the finding of NGB in astrocytes-derived exosomes [11] suggests that the exosome-mediated mechanism may be directly involved in the globin release also in breast cancer cells. However, the definition of the specific UPS involved in NGB release warrant further studies, and our research group is strongly addressing this aim.

In line with the ability of hydrogen peroxide to increase NGB secretion, the different types of UPS are largely triggered by stress [46]. For example, oxidative stress induces the release of intracellular proteins through non-classical mechanisms [48,49] and it promotes exosome secretion from cancer cells [50]. Oxidative stress is a common feature of high proliferating cancer tissue and moderate levels of ROS and in particular, H_2_O_2_, directly impact on different aspects of cancer phenotype affecting signal transduction pathways [51,52]. Indeed, during cancer transformation low/moderate levels of ROS has been demonstrated to act as signaling molecules activating MAPK/ERK1/2 and AKT pathways and contributing to cancer cell proliferation, survival, and motility [51]. On the opposite, a stronger and toxic oxidative stress would be deleterious also for cancer cells triggering, preferentially, a pro-apoptotic p38/MAPK pathway [52]. Of note, among the different signaling pathways affected by oxidative stress, evidence indicates that AKT signaling also contributes to the regulation of protein secretion through the non-classical pathway as reported for FGF-1 [53]. In this context, our present and previous data demonstrate that AKT activation plays a critical function in both E2- and oxidative stress-dependent regulation of NGB levels [17]. In particular, we previously showed that the specific activation of the PI3K/PDK/AKT pathway under E2 stimulation leads to the mitochondrial gathering of NGB whereas the activation of AKT isoforms independently from PI3K, like as it occurs after exposure to ROS-inducing compounds, mediates the NGB protein increase outside mitochondria [17]. Therefore, the activation of different AKT pathways by E2 or oxidative stress can also account for the here reported divergent effects of hormonal stimulation and hydrogen peroxide exposure on NGB extracellular release.

**Figure 6:**
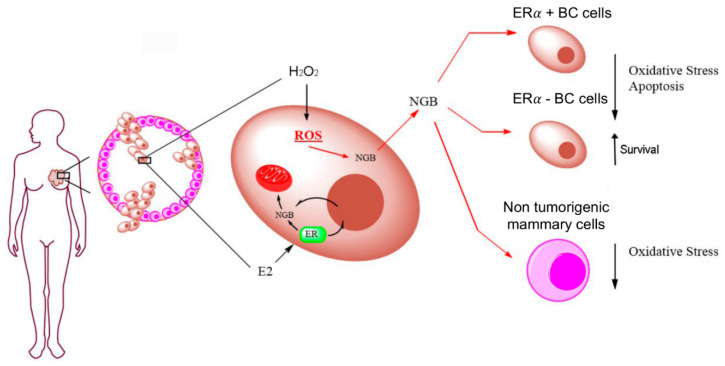
Schematic model of NGB intracellular and extracellular localization after breast cancer cells exposure to E2 or H_2_O_2_. E2 cell treatment promotes intracellular re-localization of NGB mainly at mitochondrial compartments [7,17], whereas (present results) H_2_O_2_ induces the release of NGB into the extracellular milieu where the globin can act on ERα+/− breast cancer and in non-tumorigenic epithelial mammary cells reducing oxidative stress and/or chemotherapy induced apoptosis (see text for details).

Extracellular occurrence of NGB opened the question about its functional relevance as possible autocrine/paracrine factors. For the first time, we demonstrated that sub-nanomolar and nanomolar concentrations of recombinant NGB deeply impact on breast cancer cells phenotype by increasing antioxidant response and promoting cell resistance against apoptotic cell death induced by docetaxel, a chemotherapeutic drug commonly used in clinical practice against breast cancer [24]. In addition, the effects of extracellular NGB resemble the outcomes reported for conditioned media generated from breast cancer cells treated with H_2_O_2_, but not with E2. Thus, as above reported, different NGB intracellular and extracellular localization is dependent on the nature of the stimuli (i.e., E2 vs. H_2_O_2_) and, furthermore, NGB mechanisms of action on cancer cell adaptation to oxidative stress and resistance to chemotherapeutic drugs is deeply affected by such localization. Indeed, NGB represents the key intracellular mediator of E2 antioxidant and pro-survival effects in ERα+ breast cancer cells [7,10,17]. In addition, E2-induced high intracellular NGB levels are at the root of the reduced susceptibility of ERα+ breast cancer cells to chemotherapeutic drugs [9]. On the other hand, E2 does not promote NGB extracellular release, suggesting that the globin is not necessary for the hormone-induced modification of the extracellular milieu evidenced by the CM-E2 ability to enhance transiently motility (4 h) and to reduce persistently oxidative stress (24 h) in MCF-7 cells. Contrary to E2, H_2_O_2_ promotes the accumulation of extracellular NGB that contributes to the induction of stress-adapted phenotype in those cells that do not directly face oxidative stress (Figure 6).

Of note, the effect of extracellular NGB and H_2_O_2_-induced conditioned medium show also an overlapping function on MCF-7 cell migration. In this context, molecules released after H_2_O_2_ treatment can counteract the pro-migratory function of any other factors secreted by cancer cells under resting condition and the NGB accumulation can take part in such effect. Despite the possible incongruence with evidence indicating the positive involvement of ROS and oxidant microenvironment in cancer cell migration [54,55], reported results sustain the possibility that oxidative stress-induced secretome, which comprise NGB, can preferentially favor the activation of cellular responses mainly pointed to establish the cancer cell adaptation to stress. In these regards, a phenomenon known as “dichotomy of proliferation/migration” has been indicated, which sustains that proliferation and migration may be mutualistic exclusive phenotypes [56,57]. Although the ability to evade cellular death may contribute to increase metastasis [57], it may be speculated that a similar mutual exclusion can occur, at least temporally, between stress response and migration to promote a full cancer cell adaptation to stress conditions. However, the relationship between these biological processes warrants further studies to be completely elucidated.

As a whole, our results identified that NGB is released in the extracellular milieu under oxidative stress condition, and it can behave as an inter-cellular factor able to induce stress-adapted phenotype in cancer cells and promote mechanisms of tumor resistance to therapies. Furthermore, the effects of extracellular NGB on ERα+/ERα− cancer cells and non-transformed epithelial mammary cells strongly sustain that the released globin can function in whole breast tumor environment, which commonly show a strong spatial and temporal heterogeneity in terms of cell composition [58]. These results further highlight the need to widen our vision about the role of NGB in breast cancer, also looking to its effect on the tumor microenvironment.

## 4. Materials and Methods

### 4.1. Reagents

E2, Pen-Strep solution, hydrocortisone, cholera toxin, gentamicin solution, Dulbecco′s modified Eagle medium (DMEM) with or without phenol red, protease inhibitor cocktail, bovine serum albumin fraction V (BSA), anti-Tubulin α, H_2_O_2_, Docetaxel (DTX), L-glutamine, PBS, Tris buffer, SDS, AMICON ULTRA-15 centrifugal filter unit with 3.5 KDa cut-off, and anti-NGB antibody were purchased from Merck (Darmstadt, Germany). Bradford protein assay was obtained from Bio-Rad Laboratories (Hercules, CA, USA). Anti-Bcl2, anti-AKT, and anti-pS2 antibodies were obtained from Santa Cruz Biotechnology (Santa Cruz, CA, USA). The anti-phospho-AKT (pAKT S473), anti-PARP-1 antibodies, and anti-amphiregulin (AREG) were purchased from Cell Signalling Technology Inc. (Beverly, MA, USA). The anti-Heme Oxygenase 1 (HMOX-1) was purchased from Abcam (Cambridge, UK). The chemiluminescence reagent for Western blot superpower ECL was obtained from Bio-Rad (Milan, Italy). DAPI (4′,6-Diamidino-2-Phenylindole, Dihydrochloride) was purchased from ThemoFisher Scientific (Waltham, MA, USA). Recombinant NGB (kindly gifted by Dr. Cinzia Verde, Institute of Biosciences and Bioresources, National Research Council of Italy, Naples, Italy) was obtained as described in [8] and was used at nanomolar and sub-nanomolar concentration to treat cells. All the other products were from Merck. Analytical or reagent grade products were used without further purification.

### 4.2. Cell Culture and Generation of Conditioned Medium

Human breast cancer cells MCF-7, T47D, MDA-MB-231 (ATTC, LGC Standards S.r.l., Milano, Italy) were grown in air containing 5% CO_2_ in modified, phenol red-free, DMEM medium containing 10% (*v/v*) fetal bovine serum, gentamicin (0.1 mg/mL), L-glutamine (2 mM), and Pen-strep solution (penicillin 100 U/mL and streptomycin 100 mg/mL). Non-tumorigenic epithelial mammary cells MCF-10A cells were grown in the same conditions in DMEM added with Insulin 1 μg/mL, EGF 2 ng/mL, hydrocortisone 0.05 μg/mL, and cholera toxin 0.01 μg/mL. Cell line authentication was performed periodically by amplification of multiple STR loci by BMR. Cells were treated for the indicated time with either vehicle (ethanol/PBS 1:10, v/v) or E2 (10 nM) or recombinant NGB (0.1; 1; 10 nM) or conditioned media. In experiments of apoptosis measurement, cells were pretreated with E2 (10 nM) or recombinant NGB (0.1; 1; 10 nM) or conditioned media for 4 h and then treated with Docetaxel (100 nM; 48 h).

For the generation of conditioned media breast cancer cells MCF-7 were serum starved (0%) in phenol red-free medium for 12 h. Cells were then washed twice with PBS and were cultured in fresh serum-starved medium and treated with vehicle (ethanol/PBS 1/10 *v/v*), 17-β-Estradiol (E2, 10 nM) or H_2_O_2_ (200 μM) for 30 min. After treatments, culture media were removed to avoid the residual presence of E2 or H_2_O_2_ and replaced with a fresh one for 4 h (CM 4 h) or 24 h (CM 24 h). After cell culturing (Conditioned Vehicle, CM Veh; Conditioned E2, CM E2; and Conditioned H_2_O_2_, CM H_2_O, 15 mL for each condition) were collected and, in parallel, cells were lysed in YY lysis buffer (Figure S1). CM were centrifuged at 4000 RPM for 25 min to remove death cells. Supernatants were filtered using 10 μM cut-off membrane to clean them from cell debris and then frozen at −80 °C until use for cell culturing or concentrated to a final volume of 100 μL by using AMICON ULTRA-15 centrifugal filter unit with 3.5 kDa cut-off membrane, for Western blot and ELISA analysis of secreted protein. For cell culturing, conditioned media were diluted in 1:1 ratio with fresh medium and added with 10% heat inactivated FBS.

### 4.3. Breast Cancer Tissue Section and Immunofluorescence

Surgical breast cancer paraffin-embedded sections were collected at Belcolle Hospital in Viterbo from primary tumors of patients who had underwent mastectomy. Signed informed consent was obtained from all the patients. The study was conducted in accordance with the Declaration of Helsinki, and the protocol was approved by the Ethics Committee by Belcolle Hospital and Ethics Committee Lazio 1 Protocol number 2012/CE, approval date: October 2017. Based on histopathological analysis of mastectomy, 10 ERα+, Grade 2 infiltrating ductal carcinoma were selected for the present study. For the immunofluorescence analyses, sections were de-paraffinized in xylene and rehydrated in a graded series of ethanol. For antigen retrieval, the sections were boiled in a microwave in 10 mM of sodium citrate (pH 6.0) for 10 min. Following a blocking step with 3% Bovine Serum Albumine (BSA) in Phosphate Buffered Saline (PBS) containing 0.5% of Triton-X100, the sections were incubated with the primary NGB and Collagen I antibodies o/n at 4 °C. DAPI staining were used as control of cell nuclei. After washing with PBS, sections were incubated with Alexa-Fluor 543 anti-mouse and 488 anti-rabbit secondary antibodies (Invitrogen, Carlsbad, CA, USA) for 1 h at room temperature. The slides were cover-slipped using Prolong^®^Gold anti-fade reagent (Invitrogen). Confocal analysis (40× magnification) was performed using LCS (Leica Microsystems, Wetzlar, Germany).

### 4.4. Protein Extraction and Western Blot Assay

Protein extraction and Western blot assay were performed as elsewhere reported [10]. After stimulation cells were harvested and lysed with a YY buffer mix (50 mM HEPES at pH 7.5, 10% glycerol, 150 mM NaCl, 1% Triton X-100, 1 mM EDTA, 1 mM EGTA) containing 0.70% (*w/v*) Sodium Dodecyl Sulphate (SDS). Total proteins were quantified using the Bradford Protein Assay. Solubilized proteins (15–30 μg) were resolved by 7% or 10% or 13.5% SDS-PAGE at 100 V for 1 h at 25 °C and then transferred on a nitrocellulose using the Trans-Blot Turbo Transfer System (Bio-Rad) for 7 or 12 min at 25 V. The membranes were then blocked with 5% (*w/v*) BSA in 138 mM NaCl, 25 mM Tris, pH 7.6, and 0.1 (*w/v*) Tween 20 at 25 °C for 1 h, and then incubated overnight at 4 °C with anti-NGB (final dilution 1:1000), anti Bcl-2 (final dilution 1:1000), anti-PARP-1 (final dilution 1:1000), anti-pS2 (Final dilution 1:1000), anti phospho-AKT (final dilution 1:1000), anti-total AKT (final dilution 1:1000), anti-amphiregulin (final dilution 1:1000), and anti-HMOX-1 (final dilution 1:500).

### 4.5. ELISA Sandwich

The NGB content in conditioned medium was evaluated in a final concentrated volume of conditioned media through commercially available ELISA sandwich kit (AbClonal, Woburn, MA, USA) by following manufacturer’s instructions and using a diluted series of standard NGB concentrations (eight points) for the construction of standard curve. Results are expressed as arbitrary units of the mean concentration values (pg/mL per million cells ± SD) of each condition relative to conditioned medium obtained from vehicle treated cells.

### 4.6. Quantification of ROS Levels

Cells were seeded in DMEM with phenol red and 10% of Fetal Bovine Serum. After 5 h, medium was removed and changed with a fresh one without phenol red. Depending on the experiments, cells were pretreated with E2 (10 nM) or NGB (0,1; 1; 10 nM) or conditioned media for 4 h. Then, cells were incubated for 30 min in 3 μM 2′,7′-Dichlorofluorescin diacetate (DCFH-DA) (Merck) in the dark. This probe allows the rapid quantitation of both peroxides and other ROS and reactive nitrogen species (RNS) (e.g., •OH, H2O2, ROO•, •NO). The medium was then removed, cells washed three times with serum-free medium, and then fresh medium was added containing H_2_O_2_ (400 μM) for 30 min. At the end of stimulation, cells were harvested in trypsin and analyzed using a CytoFlex (Beckman Coulter, Pasadena, CA, USA) flow cytometer. About 20,000 events/samples were analyzed for each condition. Each experiment was repeated three times.

### 4.7. Cellular DNA Content, Propidium Iodide (PI) Assay

MCF-7 cells were grown up to 80% confluence in a 96-well plate and treated with the selected compounds. The cells were fixed and permeabilized with cold Ethanol (EtOH) 70% for 15 min at −20 °C. EtOH solution was removed and the cells were incubated with propidium iodide buffer for 30 min in the dark. The solution was removed, and the cells were rinsed with PBS solution. The fluorescence was revealed (excitation wavelength: 537 nm; emission wavelength: 621 nm) with Tecan Spark 20M multimode microplate reader (Life Science by Merk Group, Darmstadt, Germany).

### 4.8. Cell Migration

To measure cell migration, trans-well chambers with 8 μM pore sized PET membranes were used. Briefly, MCF-7 cells were re-suspended in phenol-red free medium (100 μL) and loaded on the upper wells at a concentration of 15,000 cells/well. In total, 500 μL of the corresponding medium was added to the lower chamber. MCF-7 cells were re-suspended with vehicle treated medium for control condition. Where reported, recombinant NGB was added at different concentrations in cell medium, or different conditioned media were diluted in a 1:1 ratio with fresh medium before plating. After 20 h of incubation at 37 °C, migrated cells were fixed with 4% paraformaldehyde, stained with 0.1% crystal violet, and counted. Experiments were performed in quadruplicate and results are expressed as arbitrary units of the mean values (± Standard Deviation, SD) of the migrated cells relative to vehicle treated controls.

### 4.9. Statistical Analysis

The statistical analysis was performed by Student’s *t*-test or by ANOVA followed by Tukey–Kramer post-test with the PRISM 6.01 software system (GraphPad Software, Inc, San Diego, CA, USA) for Windows. In all cases, only values of *p* < 0.05 were considered significant.

## 5. Conclusions

Over the last years, a growing body of evidence indicates that the cross talk between epithelial cancer cells, non-cancerous stromal cells, and tumor microenvironment is a key determinant of cancer phenotype [59,60,61] and strategies targeting such interactions may be promising for new therapies [2,62,63,64].

In particular, it is strengthening the idea that extracellular factors including stress conditions could activate cancer and/or stromal cell responses that are not limited to intracellular rearrangements, as they may also affect the extracellular milieu and neighbor cells, favoring a tumor-promoting microenvironment [63]. Current results indicate that breast cancer cell exposure to oxidative stress induce a cell response which is spread outside, toward the tumor microenvironment and, in turn, promotes, in homotypic way, a pre-adaptation of neighbor cancer cells to stress conditions and resistance to chemotherapeutic treatment. In this context, reported data sustain a critical function of extracellular released NGB proving that (i) NGB is extracellularly released by breast cancer cells in both in vivo and in vitro conditions, (ii) H_2_O_2_ treatment promotes NGB secretion from breast cancer cells, (iii) extracellular NGB acts in reducing ROS generation under oxidative stress condition and in promoting cell resistance against chemotherapeutic treatment, and (iv) effects of extracellular NGB on breast cancer phenotype completely overlap with those reported for oxidative stress induced homotypic conditioned medium. As a whole, we shed new light on NGB indicating a new function as autocrine/paracrine factor in breast cancer. In addition, reported findings highlight the critical relationship between the nature of the stimuli and the localization-dependent functions of NGB. Indeed, two main mechanisms of action activated by endogenous E2 or stress conditions could be predicted. From one side, E2 elicits a receptor-based cell-response which modulates the intracellular NGB levels and its distribution toward mitochondria [7,17] to directly affect the phenotype of hormone-responsive cells in terms of cell adaptation to microenvironment stress condition. On the other side, the oxidative stress-induced NGB accumulation could mainly drive the globin toward the extracellular milieu to impact on neighbor cells which are not directly exposed to stress conditions, functioning as a possible “danger signal” to induce cellular pre-adaptation to stress (Figure 6). Thus, the reported effects of oxidative stress on NGB release, together with evidence indicating the presence of NGB in serum after stress conditions as ischemia/reperfusion or TBI [40,41], sustain the hypothesis that NGB extracellular-release and its inter-cellular functions can represent a common mechanism of cell response to stress insult.

Overall, the obtained data suggest that the identification of mechanisms involved in NGB release or activated by exogenous globin may open new avenue in the definition of targetable pathways for breast cancer treatment, enlarging our point of view outside the single cell response but looking at cell-cell homotypic and heterotypic communication.

## Supplementary Materials

The following are available online at https://www.mdpi.com/2072-6694/12/9/2451/s1, Figure S1: Graphic representation of experimental protocol for the generation of conditioned media (for details see Section 4), Figure S2: Whole blots images from Figure 2, Figure S3: Whole blots images from Figure 3B,C, Figure S4: Whole blots images from Figure 4B,D,F, Figure S5: Whole blots images from Figure 5C,D,G.
