# Supplementary material for: Extracellular Neuroglobin as a Stress-Induced Factor Activating Pre-Adaptation Mechanisms against Oxidative Stress and Chemotherapy-Induced Cell Death in Breast Cancer
Source: Cancers (Basel). 2020 Aug 29;12(9):2451. doi: 10.3390/cancers12092451 (PMC7564643; doi:10.3390/cancers12092451)
Supplement: Supplementary file 1 [file cancers-12-02451-s001.pdf]

Supplementary Material: Extracellular Neuroglobin as a Stress-Induced Factor Activating Pre-Adaptation Mechanisms Against Oxidative Stress and Chemotherapy-Induced Cell Death in Breast Cancer

Marco Fiocchetti, Virginia Solar Fernandez, Marco Segatto, Stefano Leone, Paolo Cercola, Annalisa Massari, Francesco Cavaliere and Maria Marino

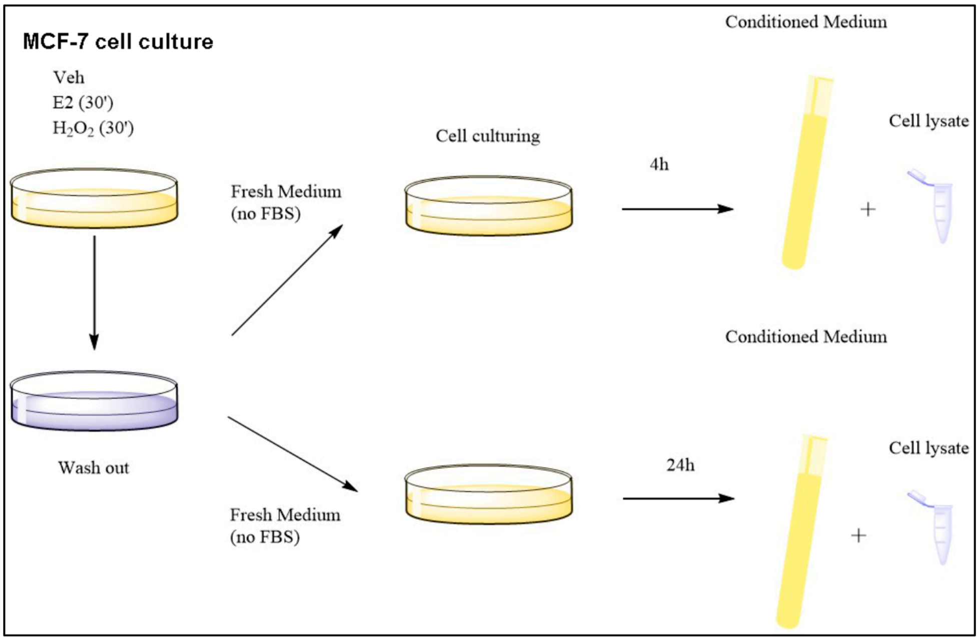

Figure S1. Graphic representation of experimental protocol for the generation of conditioned media (for details see Section 4).

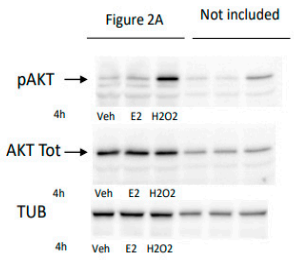

Figure 2A arbitrary units			
	pAKT	AKT TOT	TUB
Veh	11	72	79
E2	23	83	82
H2O2	94	74	73

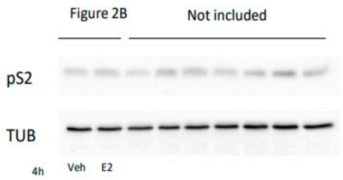

Figure 2B arbitrary units		
	pS2	TUB
Veh	15	28
E2	17	26

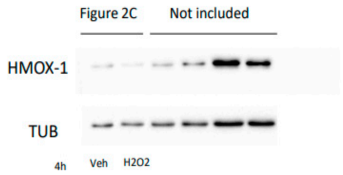

Figure 2C arbitrary units		
	HMOX-1	TUB
Veh	11	21
H2O2	7	18

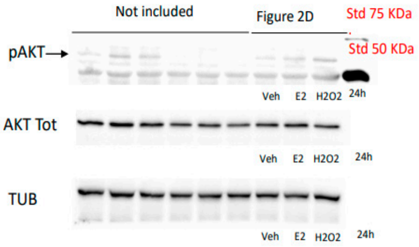

Figure 2D arbitrary units			
	pAKT	AKT TOT	TUB
Veh	6	51	74
E2	12	63	65
H2O2	19	50	95

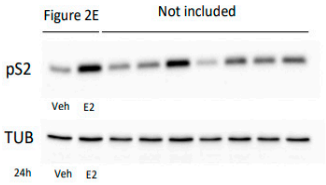

Figure 2E arbitrary units		
	pS2	TUB
Veh	8	19
E2	66	22

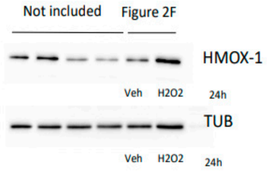

Figure 2F arbitrary units		
	HMOX-1	TUB
Veh	22	32
H2O2	70	55

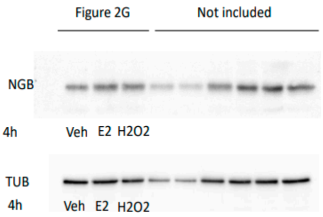

Figure 2G arbitrary units		
	NGB	TUB
Veh	24	47
E2	44	47
H2O2	35	35

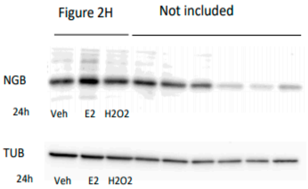

Figure 2H arbitrary units		
	NGB	TUB
Veh	40	49
E2	66	41
H2O2	44	36

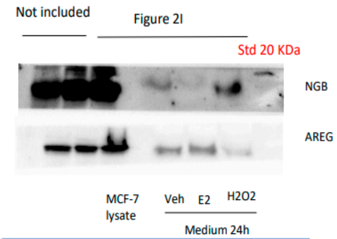

Figure 2I arbitrary units		
	NGB	AREG
MCF-7	170	59
Veh medium	18	17
E2 medium	3	22
H2O2 medium	51	4

Figure S2. Whole blots images from Figure 2.

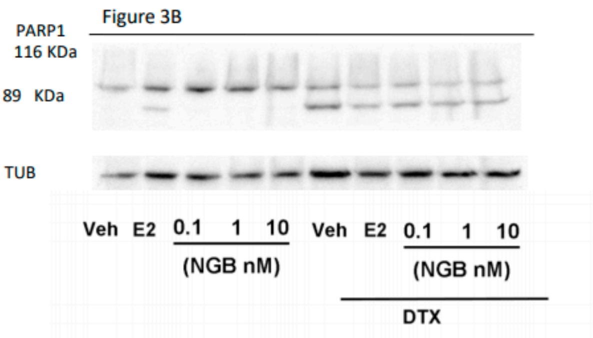

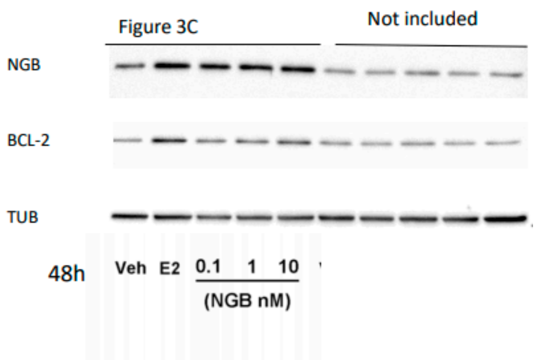

Figure 3B arbitrary units		
	PARP1 89 KDa	TUB
Veh	0	20
E2	4	49
NGB 0.1 nM	0	41
NGB 1 nM	0	32
NGB 10 nM	0.6	34
Veh+ DTX	32	81
E2+DTX	11	47
NGB 0.1 nM+DTX	15	57
NGB 1 nM+DTX	14	49
NGB 10 nM+DTX	14	50

Figure 3C arbitrary units			
	NGB	BCL-2	TUB
Veh	22	7	61
E2	57	29	64
NGB 0.1 nM	42	11	41
NGB 1 nM	48	13	50
NGB 10 nM	53	20	53

Figure 3. Whole blots images from Figure 3B,C.

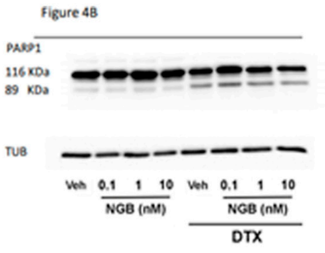

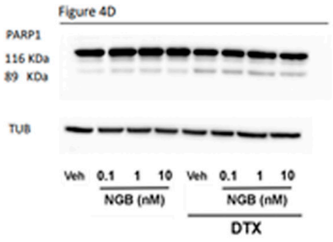

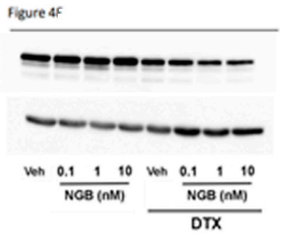

Figure 4B arbitrary units		
	PARP1 89 KDa	TUB
Veh	3	61
NGB 0.1 nM	3	46
NGB 1 nM	6	60
NGB 10 nM	3	44
Veh+ DTX	34	61
NGB 0.1 nM+DTX	51	71
NGB 1 nM+DTX	40	65
NGB 10 nM+DTX	27	75

Figure 4D arbitrary units		
	PARP1 89 KDa	TUB
Veh	1	81
NGB 0.1 nM	6	62
NGB 1 nM	5	65
NGB 10 nM	4	62
Veh+ DTX	24	64
NGB 0.1 nM+DTX	23	70
NGB 1 nM+DTX	28	85
NGB 10 nM+DTX	20	84

Figure 4F arbitrary units		
	PARP1 89 KDa	TUB
Veh	7	73
NGB 0.1 nM	7	62
NGB 1 nM	6	55
NGB 10 nM	6	62
Veh+ DTX	6	70
NGB 0.1 nM+DTX	4	103
NGB 1 nM+DTX	2	88
NGB 10 nM+DTX	3	98

Figure S4. Whole blots images from Figure 4B,D,F.

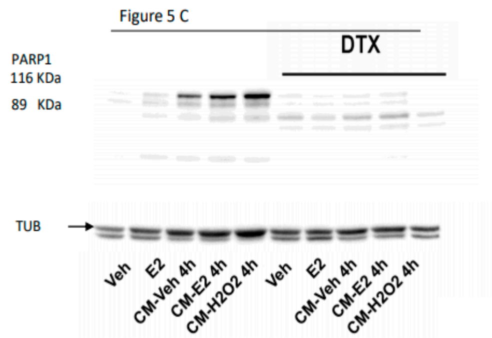

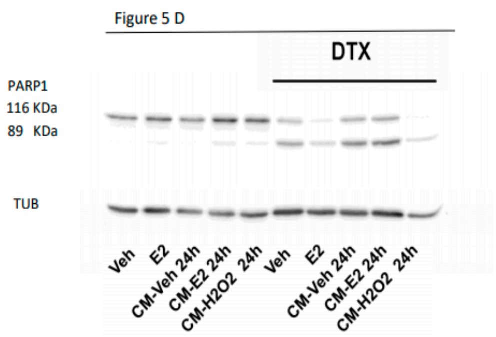

Figure 5C arbitrary units		
	PARP1 89 KDa	TUB
Veh	1	64
E2	3	96
CM-Veh 4h	5	103
CM-E2 4h	9	116
CM-H2O2 4h	10	160
DTX	15	95
DTX+E2	9	94
CM-Veh 4h +DTX	19	111
CM-E2 4h +DTX	27	111
CM-H2O2 4h +DTX	7	87

Figure 5D arbitrary units		
	PARP1 89 KDa	TUB
Veh	1	46
E2	2	52
CM-Veh 24h	1	28
CM-E2 24h	3	31
CM-H2O2 24h	2	40
DTX	17	68
DTX+E2	7	55
CM-Veh 24h +DTX	22	53
CM-E2 24h +DTX	28	71
CM-H2O2 24h +DTX	5	27

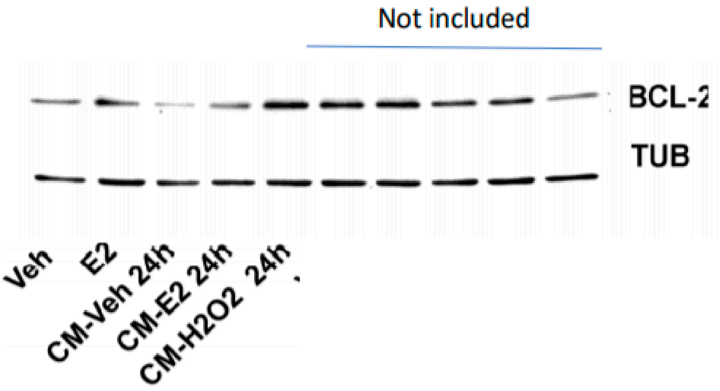

Figure 5C arbitrary units		
	Bcl-2 26 kDa	TUB
Veh	17	36
E2	46	46
CM-Veh 24h	11	36
CM-E2 24h	36	40
CM-H2O2 24h	64	59

Figure S5. Whole blots images from Figure 5C,D,G.
